# Self-reported Sleep Quality and Bone Outcomes in Older Adults: Findings from the Hertfordshire Cohort Study

**DOI:** 10.1007/s00223-020-00657-8

**Published:** 2020-01-18

**Authors:** Gregorio Bevilacqua, Hayley J. Denison, Faidra Laskou, Karen A. Jameson, Kate A. Ward, Cyrus Cooper, Elaine M. Dennison

**Affiliations:** 1 MRC Lifecourse Epidemiology Unit, University of Southampton, Southampton General Hospital, Southampton, SO16 6YD UK; 2 Centre for Public Health Research, Massey University, Wellington, New Zealand; 3 National Institute for Health Research Musculoskeletal Biomedical Research Unit, University of Oxford, Oxford, UK; 4 MRC Nutrition and Bone Health Group, Cambridge, UK; 5 Victoria University of Wellington, Wellington, New Zealand

**Keywords:** Sleep quality, Older adults, Bone microarchitecture, Computed tomography, Ageing, Bone density

## Abstract

Sleep duration may be associated with risk of osteoporosis, with suggestions that too little or indeed too much sleep may be detrimental to bone health. In this study, we considered whether perceived sleep quality is also associated with bone health in older adults. We explored this association in a cohort of 443 older community-dwelling UK adults. Sleep quality was assessed using the Pittsburgh Sleep Quality Index (PSQI); poor sleep quality was defined as > 5 on this score system. Bone density, shape and microarchitecture were assessed using dual energy X-ray absorptiometry (DXA), peripheral quantitative computed tomography (pQCT) and high-resolution pQCT (HRpQCT). Thirty-seven percent of men and 43% of women had a PSQI score greater than 5, indicative of poor perceived sleep. We found that quality of sleep was associated with altered bone microarchitecture. In men, poor sleep quality was associated with lower radial trabecular (4% slice, *p* < 0.04) and cortical (66% slice, *p* = 0.02) bone mineral density, as well as decreased tibial cortical density (*p* < 0.02) and increased porosity (*p* < 0.04), but increased size of the tibia (*p* < 0.04). In women, poor perceived sleep quality was associated with thinner (*p* < 0.03) and less dense (*p* < 0.04) cortices of the radius, but greater tibial trabecular number (*p* < 0.02) and lower separation (*p* < 0.04). Relationships with DXA parameters were non-significant after adjustment for confounders. Taking sleep medications was associated with decreased tibial size (38% and 66% slices) and strength in women (all *p* < 0.05), but not in men. Perceived sleep quality was associated with altered bone density and microarchitecture in older adults, and these differences varied according to biological sex and site. Further work is indicated to investigate possible mechanisms underlying these observations.

## Introduction

Sleep quality represents a fundamental aspect of optimal health and functioning [1]. Sleep experience can be highly subjective and related to different aspects, such as sleep onset period and frequent awakenings [2]. Sleep quality has been proven to decrease with age [3], and insufficient sleep and sleep disorder have been associated with adverse outcomes such as weight gain and obesity, diabetes, inflammation, cardiovascular disease, neurocognitive health, mental health and mortality, and these relationships have been widely discussed in the media [4]. It has also been theorised that sleep duration may be associated with risk of osteoporosis, with previous studies suggesting that insufficient or indeed excessive sleep might affect bone health in middle-aged and elderly adults [5]. However, previous studies have focussed mostly on short sleep duration [5–10] and obstructive sleep apnoea [11–16] as possible contributors to osteoporosis.

In the current study, we sought to explore, in a cohort of community-dwelling older adults in the UK, whether perceived sleep quality was associated with bone health and microarchitecture, and whether the relationships were the same in men and women.

## Methods

Participants were recruited from the Hertfordshire Cohort Study (HCS), a population-based sample of men and women born between 1931 and 1939 in Hertfordshire and originally recruited in order to study the relationship between growth in infancy and the subsequent risk of adult diseases [17, 18]. Participants completed a nurse-administered questionnaire in 1998–2002, which included details of socioeconomic status, lifestyle and diet, including calcium intake.

In 2011–2012, 443 HCS participants [222 men and 221 women; mean (SD) age 75.5 (2.5) years for men and 75.8 (2.6) for women] were visited at home by a trained fieldworker, who administered a lifestyle questionnaire. Participants were also provided with a self-administered questionnaire, which was returned to the researchers at the MRC Lifecourse Epidemiology Unit. This self-administered questionnaire included a sleep assessment tool. We assessed sleep quality using the Pittsburgh Sleep Quality Index (PSQI), a validated tool for assessing the quality of a person’s sleep; it provides a measure of sleep quality based on an individual’s evaluation of a range of sleep measures over the time period of one month [19]. The PSQI questionnaire consists of seven subcomponents: subjective sleep quality, sleep latency, sleep duration, habitual sleep efficiency, sleep disturbances, use of sleeping medication and daytime dysfunction. Each domain is weighted on a 0–3 interval scale. Answers are used to generate individual scores for each of the components, and by summing the subcomponents’ scores, a global PSQI score (ranging 0–21) is generated. A score greater than 5 is indicative of poor sleep quality and has been proven to have high sensitivity and specificity in distinguishing good and poor sleepers [19]. The PSQI questionnaire was returned by 401 (91%) of the participants (201 men and 200 women). We also assessed the prevalence of comorbid diseases in our study population. These were self-reported by participants via questionnaire, asking the question: ‘Have you been told by a doctor that you have any of the following conditions?’ The following comorbidities were recorded: hypertension, heart disease, stroke, diabetes, lung disease, thyroid disease, rheumatoid arthritis, multiple sclerosis, vitiligo, depression, Parkinson’s disease, peripheral arterial disease, osteoporosis and cancer. Social class was determined from the participants own current or most recent occupation for men and never-married women and of the husband for married women and was classified as non-manual (classes I–IIINM) or manual (classes IIIM–V) according to the 1990 OPCS Standard Occupational Classification scheme. Physical activity was calculated as the average minutes per day spent in walking, cycling, gardening, playing sport and doing house work in the last 2 weeks.

In the same year, participants attended a research clinic where the following tests were performed: a dual X-ray absorptiometry (DXA) scan (Lunar Prodigy Advanced Scanner, GE Medical Systems, UK), a peripheral quantitative computed tomography (pQCT, Stratec XCT2000, Stratec Medizintechnik, Germany) scan and a high-resolution pQCT (HRpQCT, XTreme I, Scanco, Switzerland) scan of the non-dominant radius and tibia (except when the non-dominant limb had previously fractured, in which case the dominant side was scanned). All scans were acquired by a trained technician using standard positioning techniques. Of those invited, 376 men and women agreed to participate [20].

DXA scans allowed us to measure bone mineral content (g) and areal bone mineral density (g/cm^2^) at the non-dominant hip. Positioning for all scans was completed in accordance with the manufacturer’s instructions. For pQCT scans, the radial length was measured from the distal end of the ulna styloid to the tip of the olecranon in millimetres (mm). The tibial length was measured from the prominence of the medial malleolus to the tibial plate (mm). Radial and tibial scout views identified measurement reference lines at the cortical end plates. Two slices were taken from the radial scan (4 and 66%). Four slices were taken from the tibial scan (4, 14, 38, and 66%). Trabecular parameters were measured distally (4% radius and 4% tibia) and cortical, muscle and subcutaneous fat parameters were measured in the mid-shaft (66%) for the radius and in both the mid-shaft (4% and 38%) and proximal tibia (66%). The following measurements were taken from the radius and tibia: total bone area (Tt.Ar), total mass, trabecular bone mineral density (tBMD), cortical bone mineral density (cBMD), cortical bone area (Ct.Ar), polar strength strain index (SSI) and fracture load. Short-term measurement precision error ranged from 0.88% (total tibial density, 4% slice) to 8.8% (total radial area, 66% slice), but was typically between 1 and 3%. These figures were obtained by 20 volunteers who were part of the study undergoing two scans on the same day, with limb repositioning between examinations. For all scans, a threshold of 280 mg/cm^3^ was used to separate the bone from the soft tissue background. Once separated, the default peeling algorithm was applied to the distal 4% scans to separate trabecular bone. With this peeling, 55% of the outer bone area was concentrically separated and defined as cortical and subcortical; the remaining 45% was defined as trabecular bone. For proximal scan locations, the default threshold of 710 mg/cm^3^ was used to separate cortical bone. A threshold of 280 mg/cm^3^ was used for polar SSI (mm^3^) calculation.

HRpQCT scans consist of a stack of 110 parallel CT slices using a two-dimensional detector array; the volume of interest is 9 mm in axial length with a nominal resolution (voxel size) of 82 μm. The scanned limb was immobilized with a carbon fibre cast during the examination. Antero-posterior 2D scout views were performed to determine the region to be scanned. Positioning was in keeping with the manufacturer’s guidelines and as described by Boutroy et al. [21]. Each scan was assessed for motion artefact, and, if present, a second scan was performed. A total of 40 radial images and 9 tibial images were excluded due to excessive motion artefact. Initial image analysis was carried out using the standard manufacturer’s method and Image Processing Language (IPL, Version 6.1, ScancoMedical). Further analysis was performed using an automated segmentation algorithm to obtain cortical porosity parameters. Each measure used has been validated against micro-CT imaging.

### Statistical Analysis

Descriptive statistics for continuous variables were expressed as mean and standard deviation (SD) or median and interquartile range (IQR) as appropriate. Categorical variables were expressed as frequency and percentage. Differences between men and women were assessed using Student’s *t*-tests, Mann–Whitney *U* tests, Pearson’s *χ*^2^ tests or Fisher’s exact tests, as appropriate. The bone outcomes were transformed to Fisher–Yates (FY) *z*-scores using the Fisher–Yates rank-based inverse normal transformation to normalise the data. Linear regression analyses were used to examine the associations between poor sleep quality (PSQI > 5) and the PSQI subcomponents and bone outcomes. The regression analyses were undertaken with and without adjusting for the following confounders: age, BMI, social class, smoker status (never, ex-smoker or current smoker), alcohol consumption (units per week), physical activity, dietary calcium intake [22, 23], number of comorbidities and, in women, years since menopause and hormone replacement therapy (HRT) use. The results of the regression analyses are presented as regression coefficients (*β*) and 95% confidence intervals (CI). A *p*-value of ≤ 0.05 was considered to be statistically significant. The analyses were conducted using Stata version 14.

## Results

Data on quality of sleep, BMD and bone microarchitecture were available for 323 participants, of whom 169 were men and 154 women. Table 1 provides the demographic characteristics of the participants included in the study. The mean (SD) age of participants in the study was 75.4 (2.5) years for men and 75.7 (2.6) years for women, with the mean BMI differing little between the sexes. More significant sex differences were observed in alcohol consumption, daily dietary calcium intake and smoking habits: men consumed more alcohol per week than women (median (IQR): 6.9 units (1.8–14.0) for men, 0.5 units (0.0–4.5) for women), had a higher daily dietary calcium intake than women (median (IQR): 1233 mg (1011–1413) for men, 1087 mg (913–1254) for women), and were more prone to be or have been a smoker than women, with 41.4% of them reporting to have never smoked, whereas 63.6% of women said they had never smoked. Comorbidity affected men more than women: 24% of female participants reported having no comorbid disease, while 19.5% of men said they did not have any of the conditions listed. On the other hand, more women (10.4%) than men (7.1%) reported having 4 or more concurrent diseases. Social class distribution was similar in both sexes. The majority of women (55.1%) reported never having used HRT, with only 2.7% of them saying that they were currently using HRT.

**Table 1:** Baseline characteristics of study participants by biological sex

	Men			Women			*p*-value
	*N*	Mean	SD	*N*	Mean	SD	
Age (years)	169	75.4	2.5	154	75.7	2.6	0.441
BMI (kg/m^2^)	169	27.7	3.8	152	28.3	4.9	0.174

	*N*	Median	IQR	*N*	Median	IQR	*p*-value
Activity time in last 2 weeks (min/day)	156	187	127–274	146	206	141–283	0.267
Daily dietary calcium intake (mg)	169	1233	1011–1413	154	1087	913–1254	< 0.001
Alcohol consumption (units per week)	169	6.9	1.8–14.0	154	0.5	0.0–4.5	< 0.001

	Total *N*	*N*	%	Total *N*	*N*	%	*p*-value
Smoker status	169			154			< 0.001
Never		70	41.4		98	63.6	
Ex		93	55.0		52	33.8	
Current		6	3.6		4	2.6	
Social class	162			154			0.958
I–IIINM		70	43.2		67	43.5	
IIIM–V		92	56.8		87	56.5	
No. of comorbidities	169			154			0.388
0		33	19.5		37	24.0	
1		66	39.1		45	29.2	
2		41	24.3		39	25.3	
3		17	10.1		17	11.0	
≥ 4		12	7.1		16	10.4	

	*N*	Median	IQR	*N*	Median	IQR	*p*-value
PSQI score	156	4	2–7	144	5	3–8	0.033

	Total *N*	*N*	%	Total *N*	*N*	%	*p*-value
PSQI score > 5	156	58	37.2	144	62	43.1	0.346

The median (IQR) PSQI score was 4 (2–7) for men and 5 (3–8) for women. Thirty-seven percent of men and 43% of women had a PSQI score greater than 5, indicative of poor reported sleep quality. The mean (SD) sleep duration was 6.9 (1.1) hours per night for men and women 6.9 (1.2) hours per night for women.

**Fig. 1:**
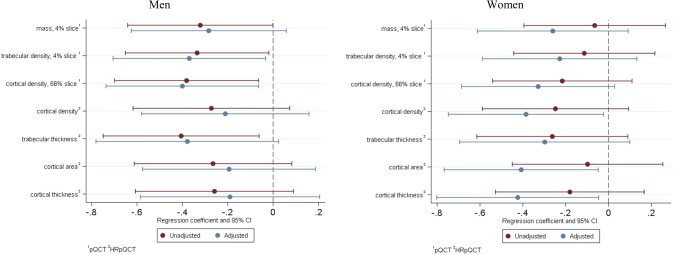
Linear regression results assessing relationship between PSQI > 5, and PQCT and HRpQCT radial bone outcomes

PSQI overall score and individual PSQI subcomponents showed no significant association with DXA-derived areal BMD, following adjustment for confounders. Conversely, relationships were found between a PSQI score > 5 and several of the bone outcomes assessed by pQCT and HRpQCT. The associations between poor quality of sleep and bone outcomes as measured by pQCT and HRpQCT for both men and women are presented in Figs. 1 (radius) and 2 (tibia). In men, poor sleep quality was associated with lower radial mass (*β* − 0.32 *z*-score, 95% CI − 0.64, − 0.00, *p* < 0.05) and tBMD (*β* − 0.34 *z*-score, 95% CI − 0.65, − 0.02, *p* < 0.05) at 4% slice, and lower radial cBMD (*β* − 0.38 *z*-score, 95% CI − 0.70, − 0.06, *p* < 0.02) at 66% slice. The associations between poor sleep and these pQCT outcomes, with the exception of radial mass at 4% slice, remained significant following adjustment for confounders. We also found associations between PSQI score > 5 and HRpQCT outcomes in men: in particular, poor sleep was associated with lower radial trabecular thickness (*β* − 0.41 *z*-score, 95% CI − 0.75, − 0.06, *p* < 0.03), higher tibial trabecular area (*β* 0.37 *z*-score, 95% CI 0.05, 0.69, *p* < 0.03) and lower tibial cBMD (*β* − 0.35 *z*-score, 95% CI − 0.67, − 0.04, *p* = 0.03) and apparent cortical thickness (*β* − 0.34 *z*-score, 95% CI − 0.66, -0.02, *p* < 0.04). Following adjustment for confounders, the associations with radial trabecular thickness and tibial apparent cortical thickness were attenuated, whilst all other associations remained significant. In addition, associations between poor sleep and higher tibial Tt.Ar (*β* 0.38 *z*-score, 95% CI 0.02, 0.73, *p* < 0.04) and cortical porosity (*β* 0.37 *z*-score, 95% CI 0.03, 0.70 *p* < 0.04) became significant after adjustment for confounders.

**Fig. 2:**
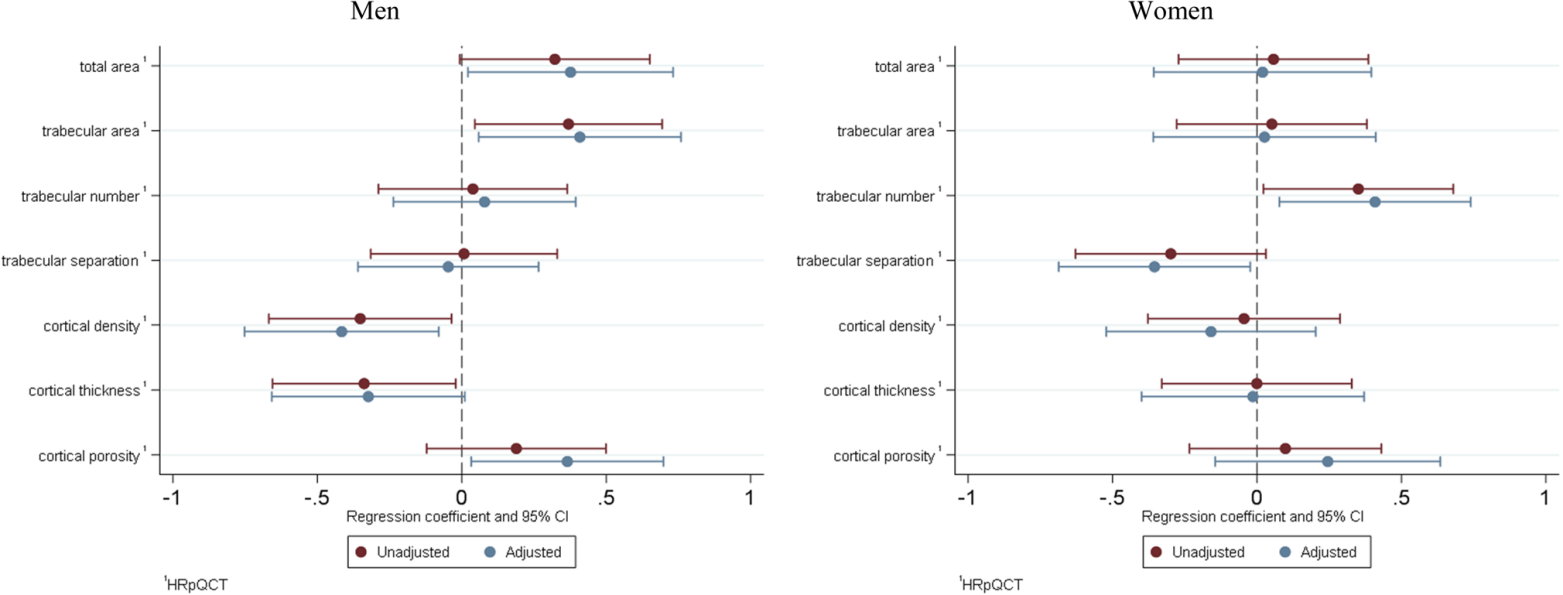
Linear regression results assessing relationship between PSQI > 5 and HRpQCT tibial bone outcomes

By contrast, in women, a PSQI score > 5 was not associated with any of the pQCT outcomes assessed. Poor sleep quality was associated with a higher tibial trabecular number (*β* 0.35 *z*-score, 95% CI 0.02, 0.68, *p* < 0.04) in women. The association persisted following adjustment for confounders, years after menopause, and HRT use. In women, a PSQI score > 5 was associated with lower radial Ct.Ar (*β* − 0.41 *z*-score, 95% CI − 0.77, − 0.05, *p* < 0.03), lower cBMD (*β* − 0.39 *z*-score, 95% CI − 0.75, − 0.02, *p* < 0.04), apparent cortical thickness (*β* − 0.42 *z*-score, 95% CI − 0.80, − 0.05, *p* < 0.03) and lower tibial trabecular separation (*β* − 0.35 *z*-score, 95% CI − 0.69, − 0.02, *p* < 0.04), only after adjustment for confounders and sex-specific confounders.

We also analysed the relationship between individual PSQI subcomponents and bone health outcomes.

### Subjective sleep quality

Poor subjective sleep quality was associated with reduced tibial polar SSI at 66% slice in men (*β* − 0.22 *z*-score, 95% CI − 0.44, − 0.00, *p* < 0.05), although the association no longer remained significant following adjustment for confounders. No further association was found in men. Conversely, PSQI poorer subjective sleep quality was associated with lower radial Tt.Ar (*β* − 0.30 *z*-score, 95% CI − 0.56, − 0.03, *p* = 0.03) and trabecular area (*β* − 0.30 *z*-score, 95% CI − 0.56, − 0.03, *p* = 0.03) at 4% slice in women (adjusted results).

### Sleep latency

Similarly, longer sleep latency did not appear to be related to bone health outcomes in men, but was associated with higher tibial trabecular number (*β* 0.28 *z*-score, 95% CI 0.10, 0.46, *p* = 0.003) and lower trabecular separation (*β* − 0.26 *z*-score, 95% CI − 0.44, − 0.08, *p* = 0.005) in women (adjusted results).

### Sleep duration

Following adjustment for confounders and sex-specific confounders, shorter sleep duration was associated with increased tibial cortical porosity in men (*β* 0.24 *z*-score, 95% CI 0.03, 0.44, *p* < 0.03) and lower tibial cBMD at 38% slice in women (*β* − 0.24 *z*-score, 95% CI − 0.44, − 0.04, *p* < 0.02).

### Sleep efficiency

Poorer habitual sleep efficiency was associated with lower radial mass (*β* − 0.16 *z*-score, 95% CI − 0.31, − 0.00, *p* < 0.05) at 4% slice in men, following adjustment for confounders. In women, poorer habitual sleep efficiency was found to be associated with higher tibial mass at 4% slice (*β* 0.19 *z*-score, 95% CI 0.05, 0.33, *p* = 0.01) and trabecular number (*β* 0.22 *z*-score, 95% CI 0.07, 0.37, *p* = 0.005), and decreased trabecular separation (*β* -0.22 *z*-score, 95% CI − 0.36, − 0.07, *p* = 0.005), following adjustment for confounders, years after menopause and HRT use.

### Sleep disturbance

In men, sleep disturbance was associated with increased radial total density (*β* 0.31 *z*-score, 95% CI 0.01, 0.61, *p* < 0.05) at 4% slice, and tibial cBMD at 38% slice (*β* 0.36 *z*-score, 95% CI 0.06, 0.65, *p* = 002) and 66% slice (*β* 0.33 *z*-score, 95% CI 0.02, 0.65, *p* < 0.04), after adjustment. In women, this PSQI domain was associated with decreased radial cortical porosity (*β* − 0.39 *z*-score, 95% CI − 0.76, − 0.03, *p* < 0.04) following adjustment for confounders and sex-specific confounders.

### Sleep medication

A higher frequency of use of sleep medications was associated with lower tibial SSI (*β* − 0.20 *z*-score, 95% CI − 0.41, − 0.00, *p* < 0.05), and Tt.Ar at 38% slice (*β* − 0.22 *z*-score, 95% CI − 0.42, − 0.01, *p* < 0.04) and 66% slice (*β* − 0.21 *z*-score, 95% CI − 0.42, − 0.00, *p* < 0.05) in women after adjustment, but no association was found in men. It must be noted that women reported a higher use of sleep medications within a month (13.8%) than men (8.9%), although the difference between sexes was not statistically significant (*p* = 0.167).

### Daytime dysfunction

No relationship was found between increased daytime dysfunction and bone health outcomes in neither men nor women, once results had been adjusted for confounders and sex-specific confounders.

## Discussion

We have found a high prevalence of poor sleep quality in a population of older community-dwelling adults, and this was consistent with the age-related deterioration of sleep found in previous studies [24]. We did not find associations between poor sleep quality (PSQI > 5) and DXA-derived areal BMD, but in contrast, we found evidence of cross-sectional associations between poor sleep quality (PSQI > 5) and altered bone density, strength and microarchitecture measured by pQCT and HRpQCT in both sexes. In general, these associations were stronger in men than in women. These observations may be explained by previous studies that suggest that body size and composition may be related to sleep parameters [25, 26]; the imperfect body size correction observed with a 2D areal technique such as DXA reinforces the use of technology that can fully adjust for body size in 3D where available. Interestingly, the direction of association appeared different at weight bearing and non-weight bearing sites. Results using pQCT scans showed significant associations in men for radial trabecular density at the epiphysis and radial cortical density at the diaphysis, whereas no association was found in women. HRpQCT imaging showed associations in men for total and trabecular areas, and cortical density and porosity at the metaphysis of the tibia, whereas in women poor reported sleep quality was associated with increased trabecular number and decreased separation at the metaphysis of the tibia, and with decreased cortical area, bone density and thickness at the metaphysis of the radius. In women, adjustment for BMI seemed to have a particular effect on the associations between metaphyseal cortical bone outcomes and poor sleep quality. It is possible that this is due to the complexity of relationship between BMI, menopause timing and sleep quality all of which would impact bone; however, such associations would need confirmation in larger cohorts. Associations between individual PSQI subcomponents and bone health outcomes also varied according to biological sex.

The association between sleep quality and bone outcomes has been previously explained as an effect of sleep deprivation on circadian rhythms and thus the consequent changes that occur in bone metabolism [27]. Disruptions in the circadian clock have indeed been linked to abnormal bone metabolism and osteoporosis [28]. In our study, bone outcomes changed according to biological sex, and this can be linked to the fact that sleep quantity and quality varies with biological sex [3]. A study by Mallampalli and colleagues found that women tend to have longer sleep latency than men, and report experiences of unrefreshing sleep and insomnia more frequently, whereas obstructive sleep apnoea is more commonly reported by men; this study also found differences in the prevalence of sleep disorders by sex, with narcolepsy and REM behaviour disorder being more common in men, and idiopathic hypersomnia being more prevalent in women [29]. In general, previous studies on sex-related differences in sleep quality report that women experience more sleep difficulties and have a higher predisposition of insomnia than men [30–33].

In our study, we found that women had a higher prevalence of poor PSQI, which is consistent with the findings of the studies mentioned above. Nonetheless, we found stronger associations between poor sleep quality and altered bone parameters in men prior to adjustments. This may reflect higher consumption of alcohol in men than women (though average intake was low in this group), or a different comorbidity profile, that could include a higher prevalence of chronic obstructive sleep apnoea, which might be a feature of obesity. Despite a significant prior literature that suggested women may report worse sleep than men, relationships appear complex. Hence, a previous study looking at sleep quantity and sleep difficulties in a population of approximately 2000 British individuals aged 16 to 93 years, despite confirming the higher prevalence of sleep difficulties in women, found that women had better self-reported sleep quality than men [34]. The population studied, however, had a significantly wider age range than our cohort, and hence, heterogeneity may mask important differences by age.

Our findings are consistent with the sex-related differences in bone microarchitecture reported by Amin and colleagues, who suggested that biological sex is associated with different bone parameters and might be site-specific [35]. In particular, the association in men between poor sleep quality and reduced tibial Tt.Ar and trabecular area (but not number), lower cBMD in both radius and tibia and increased tibial cortical porosity (but neither thickness nor area) are in line with that observed in the above-mentioned study, in which it is reported that men tend to have more trabecular thinning rather than dropout, and experience greater cortical porosity but less cortical thinning than women [35].

However, some of our results were unexpected. Previous literature has suggested that poor sleep may be detrimental to bone health. However, many studies have been performed in night workers where circadian rhythms are significantly disrupted [36–41]. Our study sample is very different; we recruited older men and women who were in retirement and where such considerations were not relevant, though comorbidity burden was higher. Additionally, we have relied on a validated self-reported sleep quality tool. It is important to recognise previous work that has suggested correlations between questionnaire scores and objective measures of sleep quantity are, at best, modest [42]. The variation in relationships by site was interesting, and may represent residual confounding, which might include some aspect of physical activity not captured by our questionnaire. In this study, physical activity was calculated as the average minutes per day spent walking, cycling, gardening, playing sport and doing house work in the last two weeks. It is possible that this approach is too granular, and an appreciation of relationships between certain types of activity to sleep and bone health would be beneficial.

We found that different subcomponents of the PSQI correlated with different pQCT and HRpQCT parameters on specific sites. Although the pattern that emerged seemed to mirror our findings from the overall PSQI score, we found more associations in women rather than men. Further refinement of the PSQI tool may be helpful to dissect relationships further. We were struck by stronger association between the use of sleep medications, and decreased bone strength and size of the tibia in women rather than men. Several central nervous system-active drugs have been associated with an increased risk of fractures [43–45], but only a few studies addressed the possible effect of such drugs on bone mineral density. A cross-sectional cohort study conducted in the US, for instance, found a correlation between decreased bone mineral density and use of anticonvulsants and opioids, but not benzodiazepines and antidepressants [46]. This study’s population was however significantly younger than ours. On the other hand, a longitudinal cohort study including more than 2000 elderly Japanese participants (mostly women) found that individuals taking angiotensin converting enzyme inhibitors or benzodiazepines experienced a higher annual loss of bone mineral density than individuals who did not take these drugs [47].

Our study has a number of strengths. HRpQCT provides measurements of bone microarchitecture and has not been previously used to explore the relationship between bone health and sleep quality. A significant strength of this study is the reasonably large sample size in a population of older adults still living in their own homes that have been extensively phenotyped and well characterised with regard to lifestyle and past medical history. Our study has also a number of limitations. The use of a self-report tool for sleep has been discussed previously, although the PSQI has been proven to be a reliable tool in differentiating good from poor sleepers [19]. The study population may not be entirely representative of the wider UK population, since all recruited participants were born in the county of Hertfordshire, were still living there in their eighth decade and were all Caucasian and therefore do not reflect the diversity of other populations in the UK. However, we have previously demonstrated that this cohort is representative of the general population with regard to anthropometric body build and lifestyle factors, such as smoking, alcohol intake and dietary calcium intake, which was in line with data found in the European Investigation into Cancer and Nutrition (EPIC) cohort [48], therefore suggesting that selection bias was minimal [17]. In addition, a ‘healthy’ responder bias is evident within the HCS [17]; however, it is unlikely to have affected the observed associations between sleep quality and bone parameters. An additional limitation of this study is the fact that sleep quality, as well as other variants, was only measured at one time point. Future studies may benefit from exploring whether longitudinal change in sleep quality is associated with change in bone health.

## Conclusion

We found that quality of sleep was associated with altered bone parameters in a cohort of older community-dwelling adults in the UK. We saw that these relationships differed according to biological sex and site: in men, poor reported sleep quality was associated, with lower tBMD and cBMD of distal and mid-shaft radius, respectively, reduced cortical density and increased cortical porosity of the tibia, but increased size of the tibial total and trabecular areas; in women, poor sleep quality was associated with cortical thinning and loss of BMD of the radius but improved trabecular parameters of the tibia. Taken together, our results suggest that self-report of poor sleep may be associated with adverse bone outcomes more so at the radius than tibia, where results were more mixed; consideration of important lifestyle factors such as alcohol consumption, physical activity and comorbidity burden is thus warranted. Distinct PSQI subcomponents seemed to have an effect on specific sites and parameters, and it is difficult to find a physiological explanation for these associations. Future studies might benefit from investigating these associations in larger populations. Use of sleep medication in women was associated with worse bone health and should be considered when reviewing older patients.
